# Single-cell multi-omics analysis reveals cellular subpopulations associated with relapse in high-risk B-ALL following intensified chemotherapy

**DOI:** 10.3389/fimmu.2025.1645546

**Published:** 2025-11-12

**Authors:** Li Liu, Xiaoyan Mao, Chunhui Yang, Na Li, Yan Zhou, Li Zhang, Nanjing Jiang, Yu Huang, Shigang Yin, Huangfan Xie, Xin Tian

**Affiliations:** 1 Institute of Advanced Biotechnology, Institute of Homeostatic Medicine, and School of Medicine, Southern University of Science and Technology, Shenzhen, China; 2 Department of Pediatrics, Children Hematological Oncology and Birth Defects Laboratory, The Affiliated Hospital of Southwest Medical University, Sichuan Clinical Research Center for Birth Defects, Luzhou, Sichuan, China; 3 Department of Hematology, Kunming Children’s Hospital, Kunming, China; 4 Pediatrics Department, Xinqi City People’s Hospital, Xinqi, Guizhou, China; 5 Department of Hematology, Sichuan Provincial Woman’s and Children’s Hospital/The Affiliated Women’s and Children’s Hospital of Chengdu Medical College, Chengdu, China; 6 Luzhou Key Laboratory of Nervous System Disease and Brain Function, Southwest Medical University, Luzhou, Sichuan, China; 7 Institute of Brain Science, Southwest Medical University, Luzhou, Sichuan, China

**Keywords:** high-risk B-ALL, scRNA-seq, scATAC-seq, PBMCs, CNV

## Abstract

**Introduction:**

Acute lymphoblastic leukemia (ALL) is the most prevalent malignant tumor in children, with B-cell ALL (B-ALL) accounting for 85% of cases. Despite advancements in chemotherapy and supportive care, a subset of high-risk B-ALL patients still experience relapse post-treatment. The molecular mechanisms underlying the relapses after intensified chemotherapy remain poorly understood.

**Methods:**

We performed an integrated single-cell multi-omics analysis combining single-cell RNA sequencing (scRNA-seq) and single-cell ATAC sequencing (scATAC-seq) on peripheral blood mononuclear cells (PBMCs) from pediatric high-risk B-ALL patients following early intensified chemotherapy, as well as from healthy controls. Bioinformatic pipelines were applied to assess cellular composition, chromatin accessibility, gene ontology enrichment, spectral clustering, and copy number variation.

**Results:**

Significant differences in cellular composition were observed between the remission and non-remission groups, with the non-remission group exhibiting a notable increase in HSC/MPP and Pro-B cells. Copy number variation (CNV) analysis also revealed that the CNV levels in HSC/MPP and Pro-B cells were higher in the non-remission group compared to other cell types. We subsequently identified a subcluster associated with resistance to intensified therapy within both the HSC/MPP and Pro-B cell groups. The drug-resistant subcluster of HSC/MPP cells was characterized by high expression of TCF4, EBF1, ERG, AL589693.1, and CRIM1, as well as enrichment of the allograft rejection pathway and the Notch signaling pathway. The drug-resistant subcluster of Pro-B cells was characterized by high expression of RPS29, B2M, RPL41, RPS21, NEIL1, AC007384.1, and CRIM1, as well as enrichment of the B cell receptor signaling pathway.

**Discussion:**

Our study identified distinct cellular subpopulations associated with treatment failure, provide insights into the molecular mechanisms underlying treatment resistance in B-ALL and may inform the development of targeted therapies for high-risk patients.

## Introduction

Acute lymphoblastic leukemia (ALL) is the most common malignant tumor in children (1). B-cell acute lymphoblastic leukemia (B-ALL), which originates from B-lineage lymphoid progenitor cells, accounts for 85% of it and is characterized by the clonal expansion of immature B lymphocytes and suppression of normal hematopoiesis. With the continuous development of combined chemotherapy, risk stratification, the combination of new drugs, and advances in supportive treatment, the prognosis for children with B-ALL is now very high, with a five-year survival rate exceeding 90% (1, 2). However, for those patients categorized as high-risk, the prognosis remains less favorable, prompting the use of early intensified chemotherapy regimens such as CAML (Cyclophosphamide, Cytosine arabinoside, 6-Mercaptopurine, and Pegaspargase) to enhance treatment outcomes. Despite these efforts, a considerable subset of patients exhibits resistance to intensified chemotherapy, resulting in subsequent relapses, with the molecular underpinnings of this resistance remaining largely enigmatic.

Single-cell RNA sequencing (scRNA-seq) and single-cell Assay for Transposase Accessible Chromatin sequencing (scATAC-seq) are powerful tools that enable a comprehensive dissection of cellular heterogeneity and chromatin accessibility at the individual cell level (3). Wang et al. have uncovered the remarkable heterogeneity of exhausted T cells in B-ALL, characterizing these subsets by their unique expression signatures by scRNA-seq (4). Granja et al., utilizing an integrative approach of scRNA-seq and scATAC-seq in the study of mixed phenotype acute leukemia, have pinpointed potential regulatory elements and transcription factors linked to leukemia-specific genes, with a particular emphasis on the crucial role of the transcription factor RUNX1 (5).These technologies provide an unprecedented opportunity to identify novel biomarkers associated with treatment response and relapse in high-risk B-ALL patients.

Although single-cell RNA sequencing has been increasing applied to study leukemia heterogeneity, the integration of transcriptomic and epigenetic single-cell data remains rare in B-ALL research. Our study uniquely combines scRNA-seq and scATAC-seq to dissert the interplay between transcriptional states and chromatin accessibility at relapse, offering a more comprehensive understanding of treatment resistance mechanisms. This dual-modality design allows us to pinpoint not only the cellular composition but also the regulatory logic underlying relapse, which has not been systematically investigated in high-risk pediatric B-ALL.

This study aims to leverage the power of scRNA-seq and scATAC-seq to delineate the molecular landscape of peripheral blood mononuclear cells (PBMCs) in high-risk B-ALL patients post early intensification treatment. We hypothesized that distinct immune subpopulations, particularly HSC/MPP and Pro-B cells, carry transcriptional and chromatin accessibility signatures that are associated with resistance to intensified chemotherapy and may serve as predictors of relapse. The identification of such biomarkers may pave the way for the development of targeted therapies and personalized treatment strategies, ultimately improving the prognosis for high-risk B-ALL patients.

## Methods

### Study design and patient cohort

Our study includes a cohort of six pediatric patients diagnosed with high-risk B-ALL who underwent early intensification treatment using the CAML protocol (**Supplementary Table S1**). High-risk B-ALL was diagnosed based on current Chinese pediatric ALL risk stratification guidelines. Peripheral blood samples were collected post-treatment, with a focus on the second round of treatment where three patients achieved remission and three experienced relapses. Additionally, peripheral blood samples from three age-matched healthy controls were obtained for comparative analysis.

### Sample collection and processing

Peripheral blood mononuclear cells (PBMCs) were isolated from the collected samples using density gradient centrifugation at 600×g for 25 minutes at 20°C. Cell number and viability were assessed by fluorescence using Reward C100 cell counter. Informed consent was obtained from all participants or their guardians, and the study was approved by the Institutional Review Board.

### Single-cell RNA sequencing

PBMCs were subjected to scRNA-seq to capture the transcriptomic landscape of individual cells. Single-cell RNA sequencing libraries were prepared using the Chromium Single Cell 3’V2 Reagent Kit, and generated using the 10x Genomic platform. All libraries were sequenced on Illumina NovaSeq 6000 platform. Each sample generated approximately 110 – 140 million raw bases, with Q30 scores above 91%. Quality control, data normalization, and dimensionality reduction were performed using established computational methods.

Upon receiving the FASTQ files from the 10x Genomics single-cell RNA sequencing results, we utilized Cell Ranger software (v7.1.0, 10x Genomics, USA) to align barcodes and UMIs to the reference genome (GRCh38), followed by filtering and quantifying gene expression at the single-cell level, resulting in a cell-gene expression matrix. Putative doublets were identified and removed using Scrublet (Scanpy pipeline) and DoubletFinder (Seurat workflow), based on the expected doublet rate from 10x Genomics specifications. This matrix was then processed using Seurat (v4.3.0, Satija Lab, USA) and Scanpy (v1.9.3, Theis Lab, Germany) (6). Cells were retained if they exhibited between 200 and 5,000 detected genes, 500 to 25,000 unique molecular identifiers (UMIs), and less than 10% mitochondrial gene expression. To further ensure data quality, doublets were identified using DoubletFinder (v2.0.3), with an expected doublet rate of 8% based on cell loading density and a pK value of 0.09. Cells with a doublet prediction score greater than 0.25 were excluded prior to downstream normalization. Cells with fewer than 200 detected genes or with mitochondrial gene read counts exceeding 10% were excluded. We integrated the data across different samples using the IntegrateLayers function through Canonical Correlation Analysis (CCA). Dimensionality reduction was performed via Principal Component Analysis (PCA), and the resulting data were clustered using UMAP. The clusters were annotated using ScType (v1.1.0, Ianevski Lab, Finland) (7), and the expression of marker genes for various immune cell types was examined. Following annotation, the R software (v4.3.1, R Foundation for Statistical Computing, Austria) was employed to calculate the proportion of each cell type across sample groups.

### Single-cell ATAC sequencing

For the single-cell ATAC-seq data, Cell Ranger ATAC (v2.1.0, 10x Genomics, USA) was used to align the reads to the reference genome and calculate the binding activity across genomic regions. The binding sites and abundance information were read using Signac (v1.9.0, Stuart Lab, USA) (8), and the genomic regions were annotated accordingly. Nuclei with 3,000 – 100,000 unique fragments, transcription start site (TSS) enrichment score ≥8, and fraction of reads in peaks (FRiP) ≥0.20 were retained. Potential doublets were identified using the ArchR addDoubletScores() function with an expected rate of 5%, and nuclei with scores >1.0 were excluded prior to downstream analysis. The distribution of binding peaks across various genomic elements was computed and statistically analyzed. Data normalization was performed, and sample integration was achieved using the Harmony algorithm (v0.1.0, Korsunsky Lab, USA), followed by UMAP clustering. To integrate single-cell transcriptomic and ATAC-seq data, the FindTransferAnchors function was used to calculate transfer anchors, and single-cell ATAC-seq data were annotated based on the single-cell RNA-seq data. The R software was then used to calculate the distribution proportions of each cell type within the single-cell ATAC-seq data.

Using the Seurat package’s FindAllMarkers function, cell-type-specific regulatory peaks were identified (9). Overrepresented motifs were found by FindMotifs function. Subsequent functional enrichment analysis of these marker genes was conducted using the ClusterProfiler (v4.6.0, Yu Lab, China) and GSVA (v1.46.0 Hanzelmann Lab, Germany) packages to identify cell types closely related to the study’s focus (10). The distribution of these motifs across cell types and genes was analyzed using single-cell ATAC-seq data.

### Data integration and analysis

The scRNA-seq and scATAC-seq data were integrated using advanced computational approaches to dissect the cellular heterogeneity and regulatory programs in high-risk B-ALL. Differential gene expressions and chromatin accessibility analysis were performed to identify markers associated with treatment response and relapse. We constructed regulatory networks by integrating scATAC-seq data with scRNA-seq data, using SCENIC to infer potential regulatory interactions between transcription factors and their target genes (11).

For subgroup analysis of HSC/MPP cells, the relevant clusters were extracted from the integrated dataset. Cells were re-normalized using SCTransform, incorporating donor ID and percentage of mitochondrial gene expression as covariates. We selected the top 1,500 highly variable genes and conducted PCA, using the first 15 principal components for UMAP visualization and Louvain clustering. Batch effect correction was applied using the Harmony algorithm, with donor ID specified as the batch variable, and performed prior to dimensionality reduction. This approach ensured that downstream clustering reflected true biological heterogeneity within the HSC/MPP compartment.

### Bioinformatic pipeline

We employed a robust bioinformatic pipeline that includes quality control, doublet detection, data normalization, dimensionality reduction using tools like PCA and t-SNE, and clustering algorithms to classify cells into distinct subtypes. Pathway enrichment analysis and gene set variation analysis (GSVA) were conducted to interpret the biological significance of the observed changes. Using the inferCNV algorithm (v1.0, Broad Institute, USA) for CNV analysis, with normal pro-B cells as references. “High CNV” refers to greater variability in chromosomal expression profiles as estimated by inferCNV, relative to reference Pro-B cells. Both gains and losses contribute to the CNV score.

All the visualizations, including UMAP plots, violin plots, heatmaps, dot plots, and bar charts, were generated using Seurat (v4.3.0), ggplot2 (v3.4.0), and ComplexHeatmap (v2.14.0) packages in R. Motif logos and accessibility tracks were plotted using ArchR and Signac.

Statistical analyses were performed in R unless otherwise stated. Comparisons between two groups were conducted using the Wilcoxon rank−sum test. For multiple comparisons, adjusted p−values were calculated using the Benjamini-Hochberg method to control the false discovery rate (FDR), with significance thresholds set at adjusted p < 0.05.

## Results

### Identification of cellular composition in B-ALL and healthy PBMC samples

To construct the landscape of cell type-specific open chromatin features and gene expression profile at the single cell level, we performed sc-RNASeq and scATAC-seq analysis on PBMCs samples from six high risk pediatric B-ALL patients with and three healthy controls (CK1, CK2, CK3) to explore their cellular composition (**Supplementary Figure S1**). PBMCs were chosen due to their accessibility and reduced invasiveness, especially in pediatric patients undergoing frequent procedures. These high-risk patients had all undergone the standard CAML (Cyclophosphamide, Cytosine arabinoside, 6-Mercaptopurine, and Pegaspargase) chemotherapy regimen. Specifically, we selected three patients who achieved complete remission (CR1, CR2, CR3) and three who experienced non-remission (NCR1, NCR2, NCR3) following two courses of intensified treatment.

**Figure 1:**
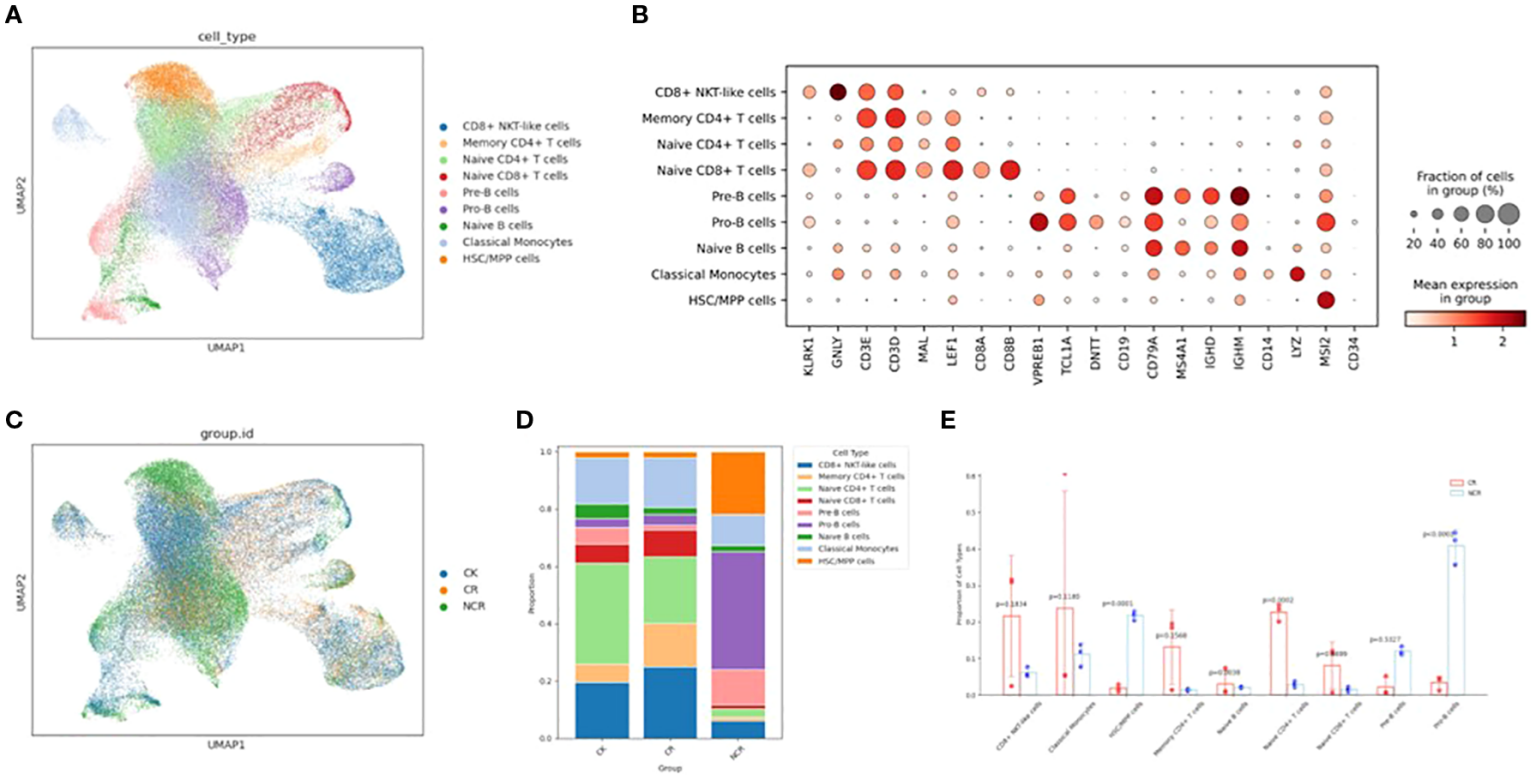
Single-cell transcriptomic analysis in ALL patient PBMC cells and healthy donor PBMC cells. **(A)** UMAP plots of nine color-coded cell clusters of all the samples. **(B)** Dot plots show the signature gene expression levels across the 9 distinct cell types. **(C)** UMAP plots of the CK, CR and NCR groups. **(D)** Relative proportion of cell clusters in each group across the 9 cellular clusters. **(E)** Histogram of the difference in cell proportion between the CR group and the NCR group. PBMC, Peripheral blood mononuclear cell; UMAP, uniform manifold approximation and projection for dimension reduction; CK, control group; CR, remission group; NCR, non-remission group.

After an initial quality control assessment and doublet removal, we obtained a total of 76458 cells, with 38773 cells from healthy donors and 37685 cells from B-ALL patients, median number of estimated cells were 6861, and the ATAC Median high-quality fragments per cell were 5071. After performing normalization and log1p transformation, highly-variable gene selection, dimensionality reduction, batch correction, and Leiden clustering, cells originating from samples were separately annotated into distinct broad cell types and visualized via Uniform Manifold Approximation and Projection (UMAP). We identified 9 main clusters in parallel according to the gene profile. These clusters were annotated as CD8+ NKT-like cells, classical monocytes, HSC/MPP cells, memory CD4+ T cells, naïve B cells, naïve CD4+ T cells, naïve CD8+ T cells, pre-B cells, pro-B cells, and progenitor cells based on the expression of known markers for each cell type (**Figures 1A, B**) (12–15).

We then analyzed the relative proportions of cell clusters (**Figures 1C, D**). The cellular composition of the ALL remission group (CR group) is similar to that of the normal group (CK), with naïve CD4 T cells and CD8+ NKT-like cells being predominant. However, the cellular composition of the non-remission group (NCR group) is significantly different from the CR group, characterized by the highest proportion of Pro-B cells (p<0.0001), a marked increase in HSC/MPP cells (p=0.0001), and a significant decrease in naïve CD4 T cells (p=0.0002) and naïve B cells(**Figure 1E**).

### Chromatin accessibility different in each cell type

**Figure 2:**
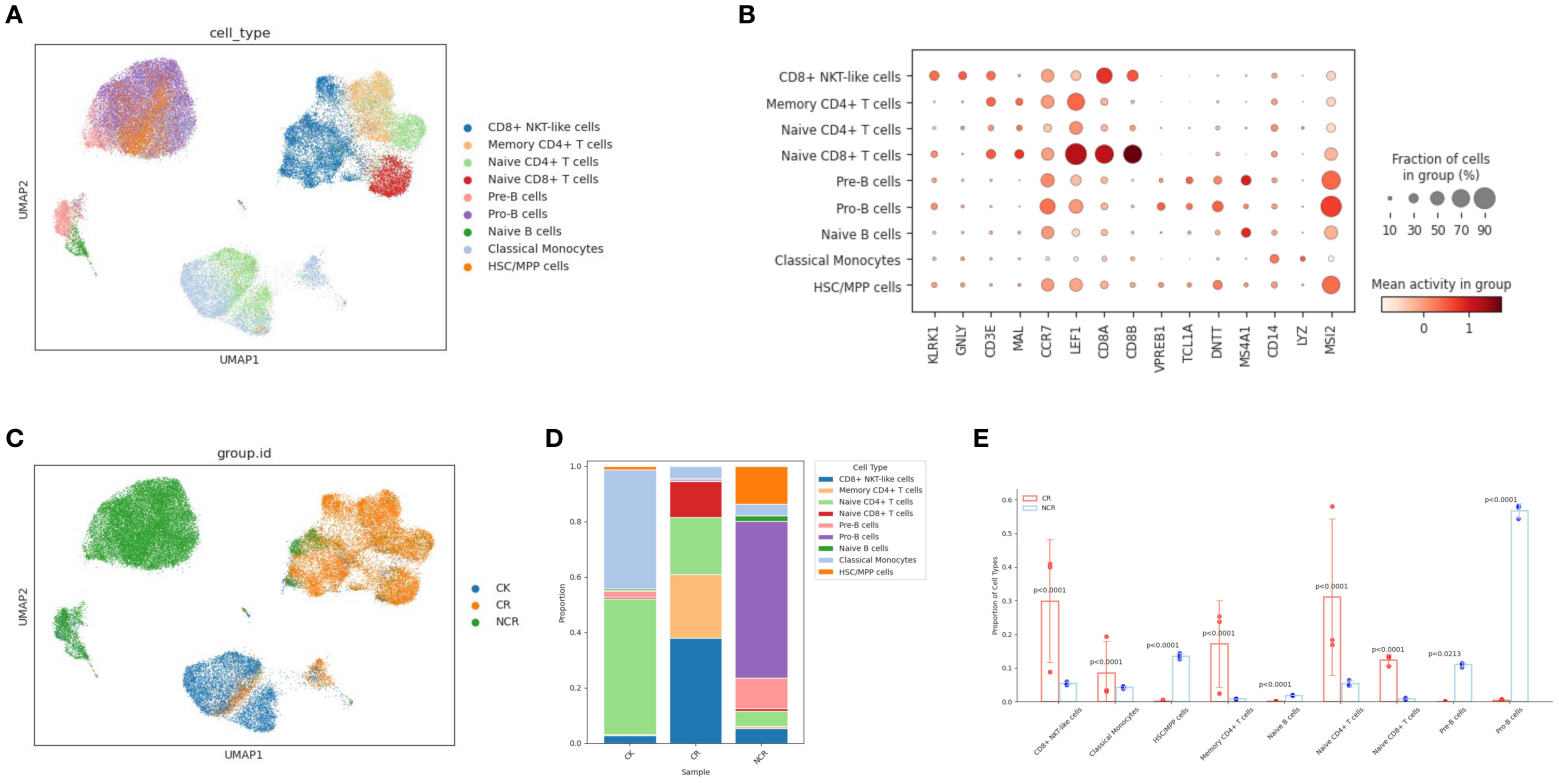
Single-cell ATAC-seq analysis in ALL patient PBMC cells and healthy donor PBMC cells. **(A)** UMAP plot visualizing clusters of PBMCs derived from scATAC-seq data. **(B)** Dot plots show the binding sites accessibility levels of representative genes across the 9 distinct cell types. **(C)** UMAP plots of the CK, CR and NCR groups. **(D)** Relative proportion of cell clusters in each group across the 9 cellular clusters. **(E)** Histogram of the difference in cell proportion between the CR group and the NCR group. PBMC, Peripheral blood mononuclear cell; UMAP, uniform manifold approximation and projection for dimension reduction; CK, control group; CR, remission group; NCR, non-remission group.

The chromatin accessibility profile of individual cells is captured by scATAC-seq. Cell-type-specific chromatin accessibility profiles are relatively unknown. So, we used Seurat’s label transfer method to predict scATAC-seq cell types based on annotated scRNA-seq data. The detailed analysis methods were executed as previously described. Comparison between scATAC-seq cell-type predictions obtained by label transfer and curated annotations of unsupervised clusters indicates that all major cell types were present in both datasets and that scATAC-seq is comparable to scRNA-seq in the detection and assignment of cell identities (**Figures 2A, B**). Subsequently, we investigated the differences in chromatin accessibility among CK, CR and NCR groups (**Figure 2C**). In the CK group, naïve CD4 T cells were predominant, followed by classical monocytes and HSC/MPP cells (**Figure 2D**). In the CR group, CD8+ NKT-like cells were dominant, which is in line with the findings from scRNA-seq. Relative to the CK group, the CR group exhibited a notable increase in the proportion of memory CD4+ T cells and CD8+ NKT-like cells, alongside a significant reduction in the proportion of naïve CD4 T cells. The cellular composition of the NCR group diverged markedly from the other groups, with Pro-B cells predominating and accounting for more than half of the cells. In contrast to the CR group, the NCR group showcased a significant upsurge in the proportion of HSC/MPP cells (p<0.0001), naïve B cells(p<0.0001) and Pro-B cells (p<0.0001), coupled with a substantial decrease in the proportion of CD8+ NKT-like cells (p<0.0001), classical monocytes (p<0.0001), memory CD4+ T cells (p<0.0001), naïve CD4 T cells (p<0.0001), and naïve CD8 T cells (p<0.0001) (**Figure 2E**).

### Gene ontology enrichment in HSC/MPP cells and Pro-B cells

**Figure 3:**
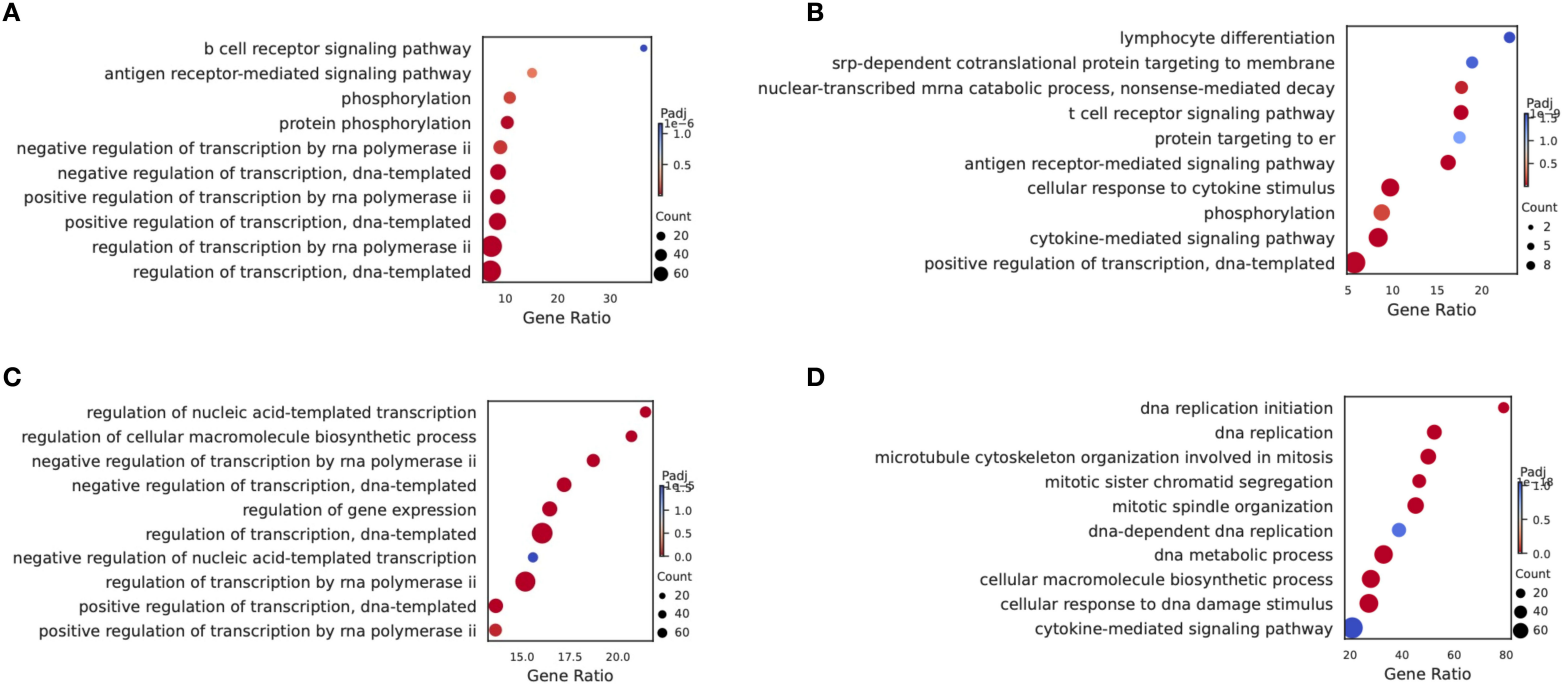
Gene ontology (GO) enrichment analysis. Compared with the CR group, the Enriched GO terms of HSCs/MPPs showed the pathway of up-regulation **(A)** and down-regulation **(B)** in the NCR group. Compared with the CR group, the Enriched GO terms of Pro-B cells showed the pathway of up-regulation **(C)** and down-regulation **(D)** in the NCR group.

Given the most pronounced differences in the relative proportions of HSC/MPP cells and Pro-B cells between the CR group and the NCR group, and the concordance of findings from scRNA-seq and scATAC-seq multi-omics analyses, we concentrated our investigation on these two specific subgroups. We analyzed the differentially expressed genes (DEGs) in HSC/MPP cells and Pro-B cells between the NCR and CR groups. The gene ontology (GO) analysis of DEGs in HSC/MPP revealed that the B cell receptor signaling pathway and antigen receptor-mediated signaling pathway were upregulated in NCR (**Figure 3A**), while lymphocyte differentiation and T cell receptor signaling pathway were downregulated in NCR (**Figure 3B**). GO analysis of DEGs in Pro-B revealed that pathways governing the regulation of transcription, gene expression, and biosynthetic processes were enhanced in the NCR group (**Figure 3C**), contrasted with a suppression of signaling pathways involved in DNA replication within the NCR group (**Figure 3D**).

### Spectral clustering of specific subtype cells (HSC/MPP and Pro-B)

**Figure 4:**
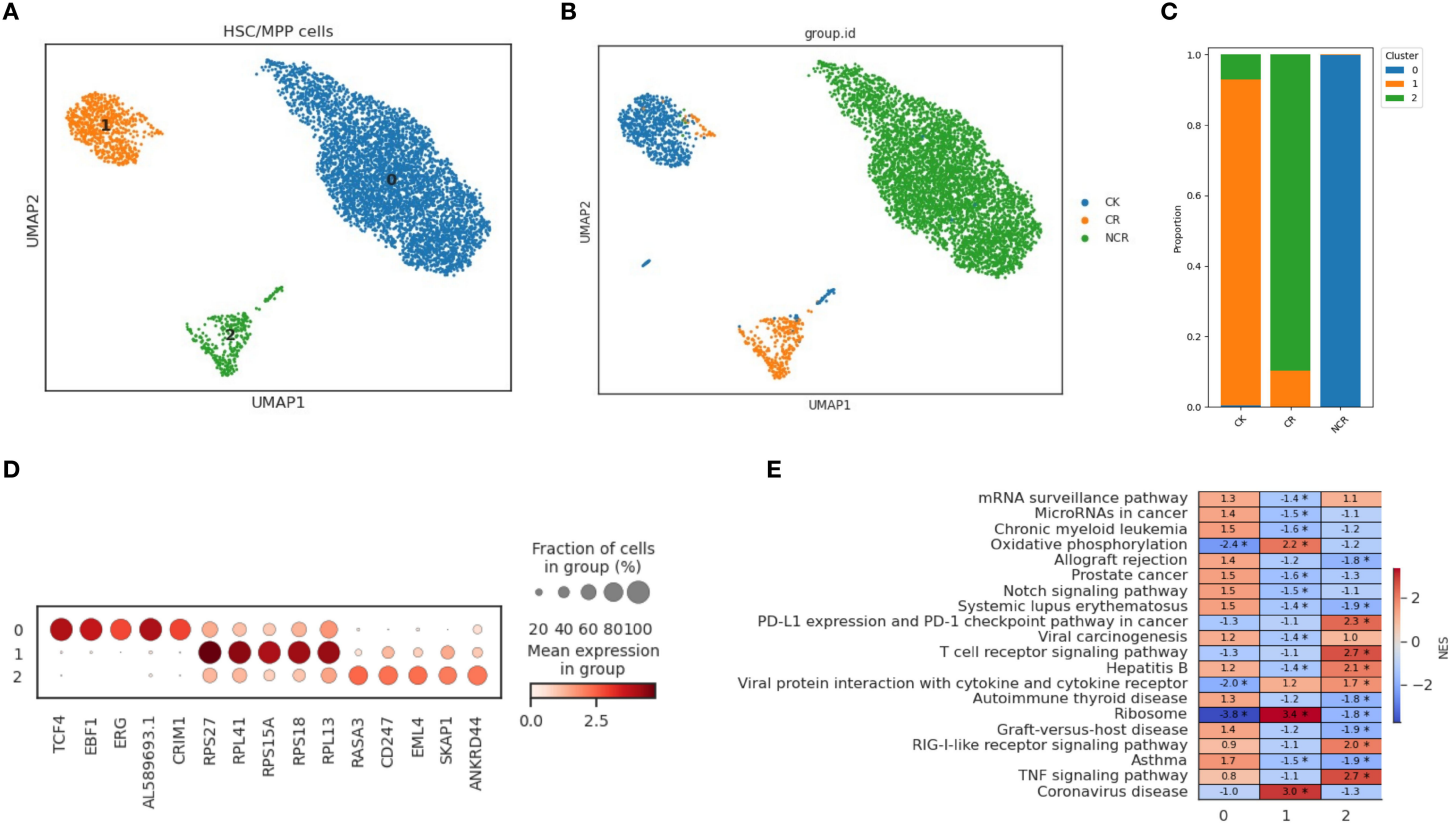
Spectral clustering of HSC/MPP cells. **(A)** Three main HSC/MPP cell subclusters were identified by UMAP analysis. **(B)** UMAP plots of HSC/MPP cells in the CK, CR and NCR groups. **(C)** The bar chart shows the proportions of each subclusters of HSC/MPP cells in the CK, CR and NCR groups. **(D)** Dot plots show the expression levels of top 5 DEGs across the three HSC/MPP subclusters. **(D)** GO analysis of DEGs in the three HSC/MPP subclusters. **(E)** Heatmap showing normalized enrichment scores (NES) of enriched pathways across HSC/MPP clusters, with color scale indicating relative pathway activity.

In order to further characterize the intrinsic structure and potential functional subtypes, we performed spectral clustering on HSCs cells using UMAP. As a result, we identified three stable subclusters in HSCs cells (**Figure 4A**). The CK group was mainly composed of subcluster 1, the CR group was predominantly made up of cells from subcluster 2, and the Pro-B cells of the NCR group were found in subcluster 0 (**Figure 4B**). Subcluster 0 included cells expressing TCF4, EBF1, ERG, AL589693.1, and CRIM1 genes (**Figure 4C**). Subcluster 1 included cells expressing genes like RPS27, RPL41, RPS15A, RPS18, and RPL13. Subcluster 2 included cells expressing genes such as RASA3, CD247, EML4, SKAP1, and ANKRD44. The TNF signaling pathway and T cell receptor signaling pathway were enriched in subcluster 2, while allograft rejection pathway and Notch signaling pathway were enriched in subcluster 0 (**Figure 4D**).

**Figure 5:**
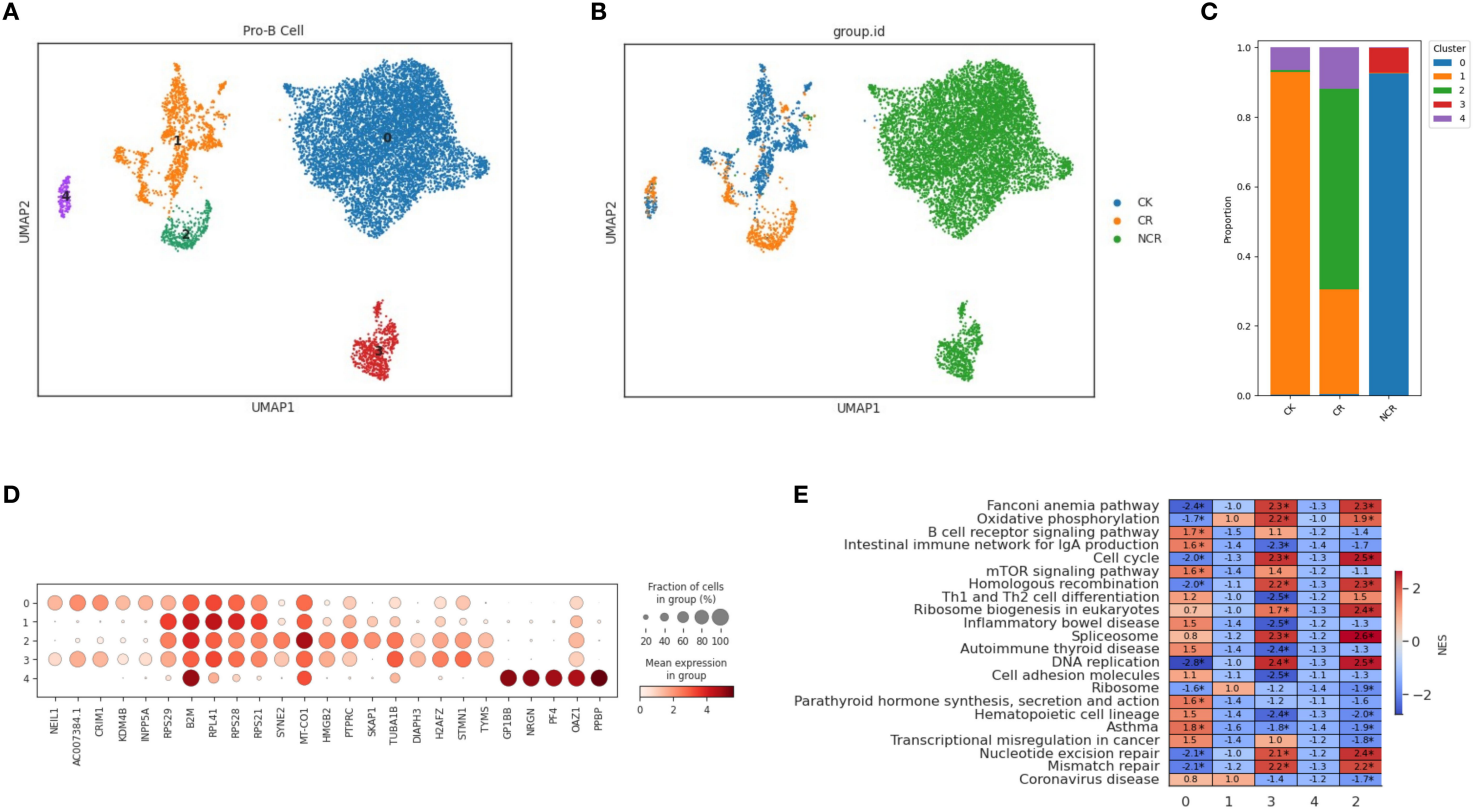
Spectral clustering of Pro-B cells. **(A)** Five main Pro-B cell subclusters were identified by UMAP analysis. **(B)** UMAP plots of Pro-B cells in the CK, CR and NCR groups. **(C)** The bar chart shows the proportions of each subclusters of Pro-B cells in the CK, CR and NCR groups. **(D)** Dot plots show the expression levels of top 5 DEGs across the three Pro-B subclusters. **(D)** GO analysis of DEGs in the five Pro-B subclusters. **(E)** Heatmap showing normalized enrichment scores (NES) of enriched pathways across Pro-B cell clusters, with color scale indicating relative pathway activity.

For the spectral clustering of Pro-B cells, five subclusters were identified (**Figure 5A**). The CK group was predominantly composed of subcluster 1, along with some cells from subcluster 4 (**Figure 5B**). The CR group was mainly made up of cells from subcluster 2, while the Pro-B cells from the NCR group belonged to subclusters 0 and 3. Each subcluster expressed its unique set of characteristic genes. The relative expression levels of cluster-specific markers in the five cellular subclusters of Pro-B have been presented in **Figure 5C**. RPS29, B2M, RPL41, and RPS21 were expressed across subclusters 0, 1, 2, and 3, but exhibited the highest expression in subcluster 1. In addition to RPS29, B2M, RPL41, and RPS21, subcluster 0 also included cells expressing the genes NEIL1, AC007384.1, and CRIM1. Subcluster 2 further included cells expressing genes such as HMGB2, PTPRC, SKAP1, and TUBA1B. Subcluster 4 was relatively distinct from the other subclusters, primarily consisting of cells expressing genes like GP1BB, NRGN, PF4, OAZ1, and PPBP. GSEA enrichment analysis showed that the B cell receptor signaling pathway was enriched in subcluster 0, while the cell cycle, DNA replication, and mismatch repair pathways were enriched in subclusters 3 and 2 (**Figure 5D**). Gene ontology enrichment analysis further revealed distinct functional programs among the HSC/MPP and Pro-B clusters (**Figures 4E, 5E**).

### Copy number variation analysis to distinguish malignant cells

**Figure 6:**
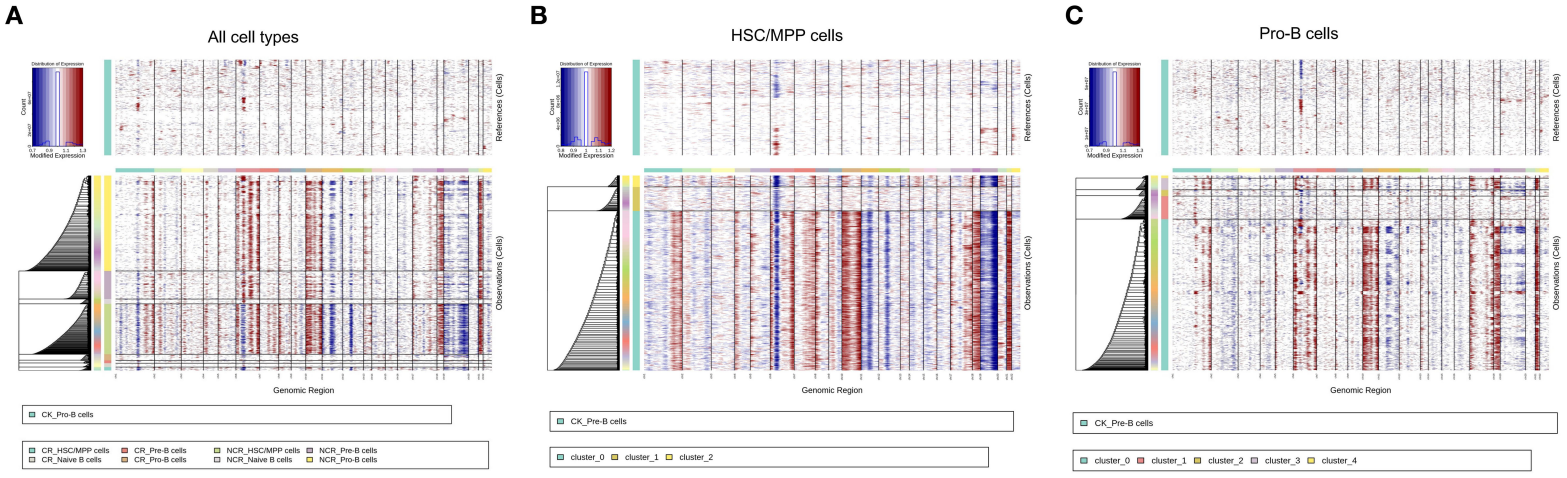
Copy number variation (CNV) analysis of all cell types **(A)**, HSC/MPP cells **(B)** and Pro-B cells **(C)**.

Copy number variation (CNV) analysis has been widely used in scRNA‐seq to investigate disease evolvement and development. To identify the cellular origin of malignant cells, we utilized the inferCNV algorithm to analyze the chromosomal CNV levels between different cell types (**Figure 6A**). As expected, the CNV levels in in the NCR group were significantly higher than those in the CR group (**Figure 6A**). Within the NCR group, HSS/MPP cells and pro-B cells exhibited higher CNV levels than other cell types.

We further analyzed the CNV levels in the three subclusters of HSC/MPP cells and the five subclusters of Pro-B cells. The results indicated that subcluster 0 of HSC/MPP cells had the highest CNV levels (**Figure 6B**), while in Pro-B cells, subgroup 0 exhibited the highest CNV levels, followed by subgroup 3 (**Figure 6C**).

### Correlation between cell-type-specific chromatin accessible degree and gene expression level in HSC/MPP cells and Pro-B cells

**Figure 7:**
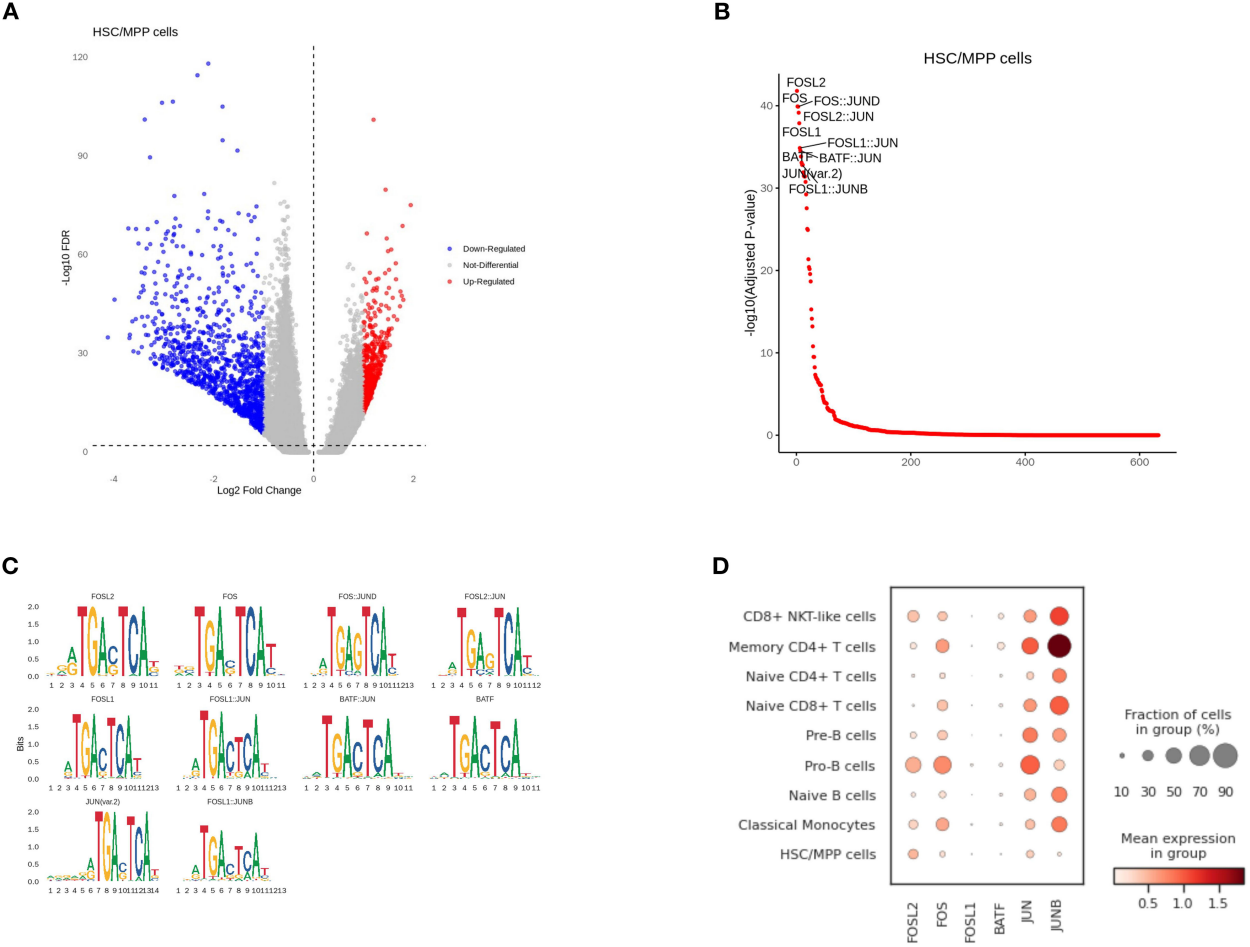
Correlation between cell-type-specific chromatin accessible degree and gene expression level in HSC/MPP cells. **(A)** Differential peaks of chromatin accessibility between HSC/MPP cells and other cell types. **(B)** Transcription factor motif analysis. **(C)** Genes with differential accessibility between HSC/MPP cells and other cell types. **(D)** The expression of representative genes in different cell types.

Compared to other cell types, HSC/MPP cells in scATAC-seq had a large number of differential peaks of chromatin accessibility (**Figure 7A**). With transcription factor motif analysis, we found that the binding sites of FOSL2, FOS, FOSL1, BATF, JUN, JUNB had higher accessibility in the HSC/MPP than in other cell types (**Figures 7B, C**). However, only FOSL2 had a significant level of expression in the HSC/MPP group, while the expression levels of other genes were relatively low. When comparing HSC/MPP cells between NCR and CR groups, no differential peaks in binding site accessibility were observed (**Figure 7B**). Furthermore, expression patterns of representative immune-related genes across cell types were also assessed (**Figure 7D**).

**Figure 8:**
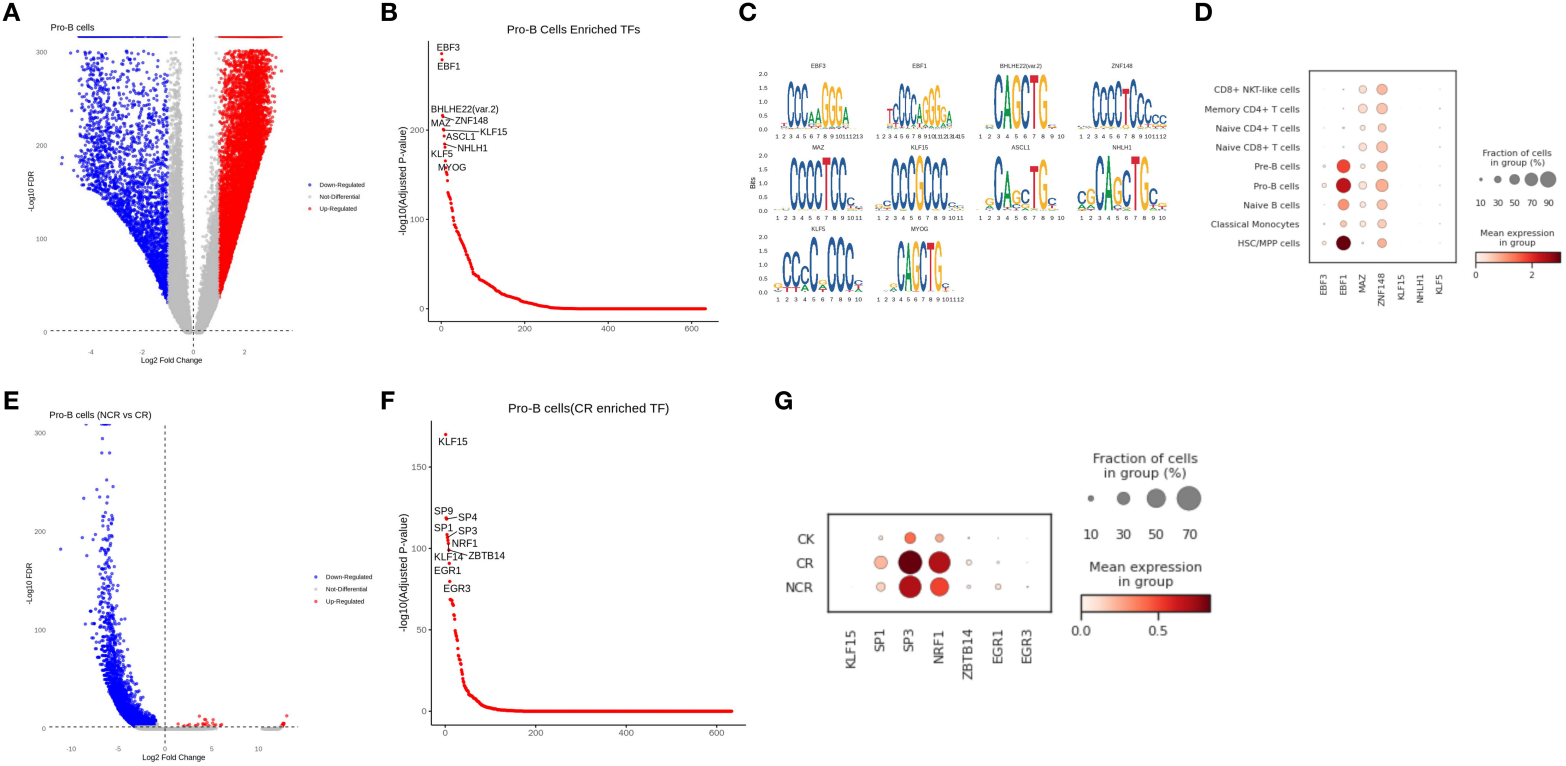
Correlation between cell-type-specific chromatin accessible degree and gene expression level in Pro-B cells. **(A)** Differential peaks of chromatin accessibility between Pro-B cells and other cell types. **(B)** Transcription factor motif analysis in Pro-B cells. **(C)** Genes with differential accessibility between Pro-B cells and other cell types. **(D)** The expression of representative genes in different cell types. **(E)** Differential peaks of chromatin accessibility in Pro-B cells between non-remission group and remission group. **(F)** Transcription factor motif analysis of Pro-B cells in remission group. **(G)** The expression of representative genes in normal, remission and non-remission group.

The analysis of the Pro-B subgroup in scATAC-seq showed that the binding sites of EBF3, EBF1, MAZ, ZNF148, KLF15, NHLH1, and KLF5 had higher accessibility in Pro-B cells than in other cell types (**Figures 8A-C**). We further analyzed the expression differences of these genes in scRNA-seq and found that EBF1 and ZNF148 were highly expressed in Pro-B cells (**Figure 8D**). When comparing Pro-B cells between NCR and CR groups, the differential peaks in NCR were mainly downregulated, which means they were predominantly enriched in the CR group (**Figure 8E**). We found that the accessibility of KLF15, SP1, SP3, NRF1, ZBTB14, EGR1, EGR3 binding sites in Pro-B was higher in the CR group than in the NCR groups (**Figure 8F**). Among them, SP1, SP3, NRF1 also showed higher expression levels in scRNA-seq in the CR group compared to the NCR group, which is consistent with scATAC-seq (**Figure 8G**).

## Discussion

The standard intensive therapy for high-risk B-ALL patients currently involves two cycles of the CAML protocol. Despite this, some patients exhibit resistance to early intensified treatment, resulting in therapeutic failure. However, our understanding of the molecular mechanisms underlying chemotherapy sensitivity and the identification of patients who are likely to benefit from such treatments remains limited. Literature has indicated that infants with CD10-positive blasts, in the absence of 11q23/MLL rearrangements, tend to have improved outcomes and respond better to intensified therapy (16). Infants with B-ALL harboring germline MLL genes show superior responses to chemotherapy compared to those with rearranged MLL genes, achieving a 5-year event-free survival rate of 95.5% with manageable treatment toxicity (17). Traditional diagnostic methods, which assess the average mutations and expressions across a population of cells, can obscure the signals of cellular heterogeneity, particularly overlooking the signals from rare cell types. To address this, we employed single-cell RNA sequencing (scRNA-seq) combined with single-cell ATAC sequencing (scATAC-seq) to analyze differential gene expressions in specific cellular subsets within peripheral blood mononuclear cells (PBMCs) from B-ALL patients who experienced remission or relapse following intensive therapy, as well as from healthy controls. By clustering these expression profiles, we inferred genes associated with relapse within particular cell types.

The scRNA-seq results elucidate that the cellular composition of the remission group is similar to that of the normal group, which also demonstrates the effectiveness of the treatment at the cellular level. However, the cellular composition of the non-remission group is significantly different from the other groups, with the most pronounced changes observed in the HSC/MPP and Pro-B cell populations. In both the remission and normal groups, the proportions of these two types of cells are relatively low, whereas in the non-remission group, the proportions of these cells have increased dramatically, becoming the predominant types. This may be due to the failure of intensified therapy, where normal hematopoiesis in leukemia continues to be suppressed, leading to anemia, thrombocytopenia, and neutropenia. The body compensates by increasing the number of HSC/MPP cells, which possess multilineage differentiation and self-renewal capabilities (18). Simultaneously, there is an arrest of B cell development at the Pro-B stage, impeding cellular maturation and precipitating the accumulation of these immature cell forms.

Compared to the scRNA-seq analysis, the scATAC-seq analysis reveals even greater differences in cellular proportions among the normal, remission, and non-remission groups, possibly because the epigenome is more prone to change than the transcriptome. In the non-remission group, the cell proportions based on chromatin accessibility also show that HSC/MPP and Pro-B cells are the most predominant, which is consistent with the scRNA-seq findings. CNV analysis also demonstrates that the CNV levels in HSC/MPP and Pro-B cells are higher in the NCR group than in other cell types, indicating that malignant cells in PBMCs are primarily concentrated in these two cell populations. Therefore, we focus our analysis on these two subgroups. CNV differences observed between CR and NCR groups may reflect individual genomic background rather than therapy-specific changes. We interpret CNV variation as a correlate of clonal genomic instability, but not direct proof of malignancy.

Further stratification of HSC/MPP identified three distinct subclusters, with the normal, remission, and non-remission groups each corresponding to different subclusters. The HSC/MPP cells from the NCR group are predominantly found in subcluster 0, which shows high expression of TCF4, EBF1, ERG, AL589693.1, and CRIM1, along with enrichment of the allograft rejection pathway and the Notch signaling pathway. These associations suggest that this subpopulation may be involved in resistance to intensified therapy. However, these findings are observational and require functional validation to determine any causal role. TCF4 is a transcription factor belonging to the basic helix-loop-helix (bHLH) family, which is involved in lymphoid cell differentiation (19). In B-ALL, TCF4 may linked in the regulation of B cell proliferation and differentiation, as well as the self-renewal of leukemic stem cells. EBF1 is a key transcription factor in B cell development, essential for the proliferation and maturation of early B cells (20). Its aberrant expression may be associated with the proliferation and survival of leukemia cells (21). ERG is a member of the ETS family, involved in multiple cellular processes, including cell proliferation, differentiation, and migration (22). In B-ALL, ERG may be involved in the migration and invasion of leukemic cells, as well as resistance to therapy. CRIM1 is a transmembrane protein that modulates the bone morphogenetic protein (BMP) signaling pathway, which plays a role in various cellular processes, including cell differentiation and proliferation (23, 24). It may be involved in regulating the differentiation of leukemia cells and their response to treatment. Current research on the biology of ALL has identified several recurrent ALLs with targetable pathways, including the Notch signaling pathway (25). The upregulation of the Notch signaling pathway in subcluster 0 of HSC/MPP cells suggests a possible role in relapse biology, but functional studies are necessary to determine whether this pathway is directly involved in therapy resistance.

In addition to its role in hematopoietic development and leukemogenesis, Notch signaling has been implicated in immune modulation and resistance to immunotherapy (26). Studies in T-cell acute lymphoblastic leukemia (T-ALL) and other malignancies have shown that aberrant Notch activation may suppress T cell activation, promote an immunosuppressive tumor microenvironment, or alter immune checkpoint expression (27, 28). These mechanisms may contribute to poor responses to immunotherapies such as CAR-T cells or immune checkpoint inhibitors. Although our study did not directly examine immunotherapy response, the enrichment of Notch signaling in the NCR-associated HSC/MPP subpopulation raises the possibility that this pathway may influence immune escape and warrants further investigation in the context of relapsed B-ALL.

Pro-B cells were stratified into 5 subclusters through spectral clustering, with the NCR group corresponding to subclusters 0 and 3, among which the majority of the pro-B cells in NCR were in subcluster 0. This subcluster is characterized by high expression of RPS29, B2M, RPL41, RPS21, NEIL1, AC007384.1, and CRIM1, as well as enrichment of the B cell receptor signaling pathway. Previous studies have shown that treatment with glucocorticoids leads to enrichment of the B cell receptor signaling pathway in B-ALL cells, and that B cell development is crucial for glucocorticoid resistance in B-ALL cells (29). Therefore, the upregulation of the B cell receptor signaling pathway in subcluster 0 of Pro-B cells may also contribute to the patients’ resistance to intensified chemotherapy.

Due to the clinical constraints and ethical consideration, the sample size in our study is limited and not longitudinally paired. As a result, caution should be exercised in extrapolating our findings, and larger, paired cohort studies will be necessary to validate the identified subpopulations and mechanisms. Future validations are needed. First, the potential pathogenic genes identified have not yet undergone functional validation and require further functional experiments for confirmation in the future. Secondly, although these genes may all be related to the relapse of intensified therapy in B-ALL, our sample size is small, and a larger sample is needed to verify the universality and specificity of these relapse genes in specific cell types. Thirdly, our study is limited by the absence of full molecular subtype classification for each B-ALL patient, which poses a challenge given the extensive heterogeneity of the disease. Over 20 subtypes have been recognized, many of which are associated with distinct prognoses and treatment responses. While our inferCNV-based approach provides insights into global genomic instability, it may not detect subtype defined by gene fusion or single-nucleotide variants. Fourthly, all single-cell data in this study were derived from peripheral blood mononuclear cells (PBMCs), rather than from the bone marrow microenvironment, which is the primary site of leukemic development in B-ALL. While PBMCs provide a minimally invasive snapshot of systemic immune and leukemic cell states, they may not fully capture the complexity of interactions occurring in the bone marrow niche. Future studies involving bone marrow samples will be essential to validate and extend these findings.

Although in the absence of *in vitro* validation, our conclusions are supported by consistency with previous studies. The relapse-associated HSC/MPP subcluster enriched for *TCF4*, *EBF1*, and *ERG* expression, together with Notch signaling, aligns with reports implicating these factors in B-cell development, leukemic stemness, and therapy resistance (20–22). Similarly, the Pro-B subcluster characterized by *RPS29*, *B2M*, and enrichment of the BCR pathway corresponds with published evidence linking BCR signaling to glucocorticoid resistance in B-ALL (26, 28). Prior studies have also emphasized the role of CRIM1 and BMP signaling in leukemic differentiation and drug response (23, 24, 29). Taken together, these consistencies with the literature reinforce the robustness of our multi-omics findings, while also highlighting Notch and BCR signaling as promising axes for future mechanistic and therapeutic exploration.

In conclusion, our study has identified genes within specific cellular subsets that are associated with the failure of intensified therapy in B-ALL, thereby enriching the existing knowledge base for the investigation of drug resistance mechanisms in B-ALL. Additionally, we posit that the disruption of signaling pathways due to aberrant expression of these genes may be a significant contributor to therapeutic resistance. Consequently, the development of targeted inhibitors against these genes and their associated pathways could potentially overcome drug resistance in B-ALL, thereby enhancing therapeutic outcomes and providing increased benefits to patients.

## Data Availability

The original contributions presented in the study are publicly available. This data can be found here: https://www.ncbi.nlm.nih.gov/ accession number PRJNA1333439 and PRJNA1336723.

## Supplementary material

Supplementary Table S1 Clinical and demographic information of all patient samples used in this study.

Supplementary Figure S1 Schematic overview of the study workflow. Peripheral blood samples were collected from three CR (complete remission), three NCR (non-complete remission), and three healthy control children. After mixing with PBS/EDTA, samples underwent density gradient centrifugation to separate plasma, red blood cells, and PBMCs. PBMCs were subjected to 10x Genomics-based 3′ single-cell RNA sequencing and ATAC sequencing. Downstream analyses focused on identifying and comparing HSC/MPP and Pro-B cell populations across the three groups to explore immune remodeling and recurrence mechanisms in high-risk B-ALL.
