# Correction: Single-cell multi-omics analysis reveals cellular subpopulations associated with relapse in high-risk B-ALL following intensified chemotherapy

**DOI:** 10.3389/fimmu.2026.1836508

**Published:** 2026-04-09

**Authors:** Li Liu, Xiaoyan Mao, Chunhui Yang, Na Li, Yan Zhou, Li Zhang, Nanjing Jiang, Yu Huang, Shigang Yin, Huangfan Xie, Xin Tian

**Affiliations:** 1 Institute of Advanced Biotechnology, Institute of Homeostatic Medicine, and School of Medicine, Southern University of Science and Technology, Shenzhen, China; 2 Department of Pediatrics, Children Hematological Oncology and Birth Defects Laboratory, The Affiliated Hospital of Southwest Medical University, Sichuan Clinical Research Center for Birth Defects, Luzhou, Sichuan, China; 3 Department of Hematology, Kunming Children’s Hospital, Kunming, China; 4 Pediatrics Department, Xinqi City People’s Hospital, Xinqi, Guizhou, China; 5 Department of Hematology, Sichuan Provincial Woman’s and Children’s Hospital/The Affiliated Women’s and Children’s Hospital of Chengdu Medical College, Chengdu, China; 6 Luzhou Key Laboratory of Nervous System Disease and Brain Function, Southwest Medical University, Luzhou, Sichuan, China; 7 Institute of Brain Science, Southwest Medical University, Luzhou, Sichuan, China

**Keywords:** high-risk B-ALL, scRNA-seq, scATAC-seq, PBMCs, CNV

In the published article, there was an error in affiliation(s) 1 and 5. For affiliation 1, instead of “Department of Pediatrics, Qujing Medical College, Qujing, China”, it should be deleted. For affiliation 5, instead of “Department of Pediatrics, Da Li University, Da Li, China”, it should be “Pediatrics Department, Xinqi City People’s Hospital, Guizhou Province”.

Affiliation “Institute of Brain Science, Southwest Medical University, Luzhou, Sichuan, China” was omitted for authors “Shigang Yin and Huangfan Xie”. This affiliation has now been added and the affiliations have been reordered to conform to the previous affiliation 1's removal.

The original article has been updated.

