# Supplementary material for: Single-cell multi-omics analysis reveals cellular subpopulations associated with relapse in high-risk B-ALL following intensified chemotherapy
Source: Front Immunol. 2025 Nov 12;16:1645546. doi: 10.3389/fimmu.2025.1645546 (PMC12648095; doi:10.3389/fimmu.2025.1645546)
Supplement: Supplementary Table S1 — Clinical and demographic information of all patient samples used in this study. [file Table1.docx]

| Patient | Group | Age/years | Sex | Molecular Features of B-ALL |
| --- | --- | --- | --- | --- |
| 1 | CR | 10 | Male | IKZF1^+^ |
| 2 | CR | 13.3 | Male | BCR-ABL^+^, p210 fusion protein |
| 3 | CR | 11 | Female | MLL^+^, TP53^+^. PIK3R1^+^ |
| 4 | NCR | 4.5 | Male | NRAS, ATM/TP53 |
| 5 | NCR | 8 | Male | TCF3/ZNF384^+^ |
| 6 | NCR | 11.4 | Male | BCR-ABL P190, Ph^+^ |
