# Supplementary figures and images for: Single-cell multi-omics analysis reveals cellular subpopulations associated with relapse in high-risk B-ALL following intensified chemotherapy
Source: Front Immunol. 2025 Nov 12;16:1645546. doi: 10.3389/fimmu.2025.1645546 (PMC12648095; doi:10.3389/fimmu.2025.1645546)

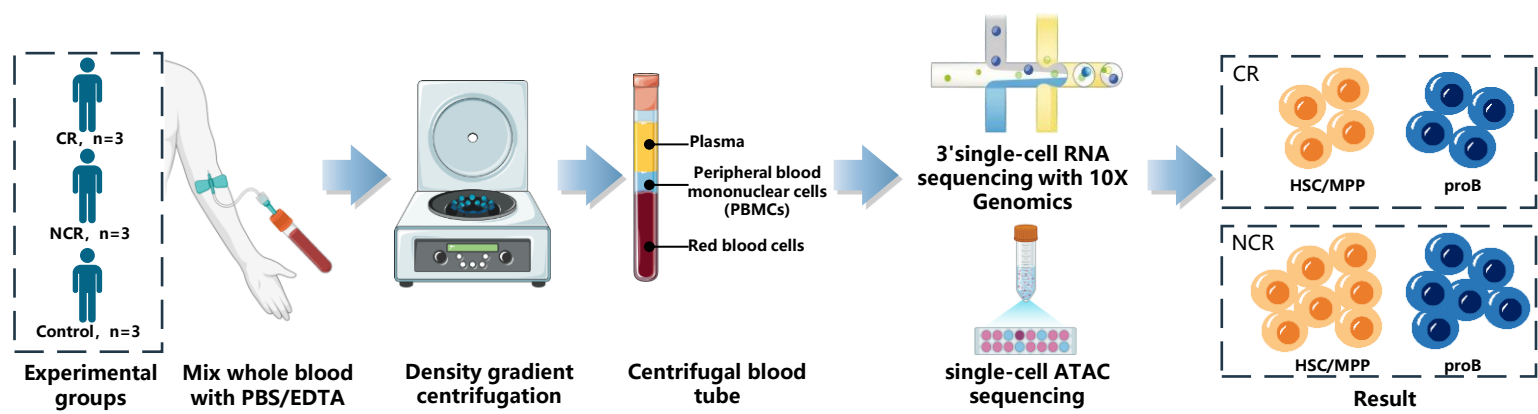

Supplement: Supplementary Figure S1 — Schematic overview of the study workflow. Peripheral blood samples were collected from three CR (complete remission), three NCR (non-complete remission), and three healthy control children. After mixing with PBS/EDTA, samples underwent density gradient centrifugation to separate plasma, red blood cells, and PBMCs. PBMCs were subjected to 10x Genomics-based 3′ single-cell RNA sequencing and ATAC sequencing. Downstream analyses focused on identifying and comparing HSC/MPP and Pro-B cell populations across the three groups to explore immune remodeling and recurrence mechanisms in high-risk B-ALL. [file DataSheet1.pdf]
